# Supplementary figures and images for: Optimising sampling of fish assemblages on intertidal reefs using remote underwater video
Source: PeerJ. 2023 May 22;11:e15426. doi: 10.7717/peerj.15426 (PMC10211360; doi:10.7717/peerj.15426)

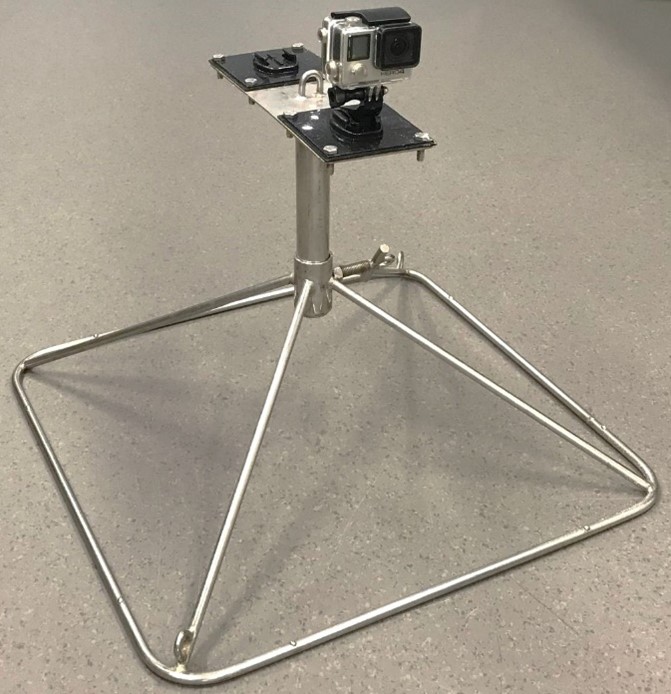

Supplement: Supplemental Information 2 — GoPro Hero 4 camera mounted on metal frame. [file peerj-11-15426-s002.jpg]
