# Supplementary material for: Optimising sampling of fish assemblages on intertidal reefs using remote underwater video
Source: PeerJ. 2023 May 22;11:e15426. doi: 10.7717/peerj.15426 (PMC10211360; doi:10.7717/peerj.15426)
Supplement: Supplemental Information 3 [file peerj-11-15426-s003.docx]

| **Contrast** | **estimate** | **SE** | **df** | **t.ratio** | **p.value** |
| --- | --- | --- | --- | --- | --- |
| i12 MaxNT - i12 MeanCountT | 2.023 | 0.555 | 431 | 3.645 | **0.053** |
| i12 MaxNT - i12 SpeciesRichness | -0.111 | 0.555 | 431 | -0.201 | 1.000 |
| i15 MaxNT - i15 MeanCountT | 2.271 | 0.555 | 431 | 4.091 | **0.011** |
| i15 MaxNT - i15 SpeciesRichness | 0.043 | 0.555 | 431 | 0.078 | 1.000 |
| i20 MaxNT - i20 MeanCountT | 2.234 | 0.555 | 431 | 4.025 | **0.014** |
| i20 MaxNT - i20 SpeciesRichness | -0.091 | 0.555 | 431 | -0.164 | 1.000 |
| i30 MaxNT - i30 MeanCountT | 2.561 | 0.555 | 431 | 4.614 | **0.001** |
| i30 MaxNT - i30 SpeciesRichness | -0.129 | 0.555 | 431 | -0.232 | 1.000 |
| i60 MaxNT - i60 MeanCountT | 2.325 | 0.555 | 431 | 4.189 | **0.008** |
| i60 MaxNT - i60 SpeciesRichness | -0.010 | 0.555 | 431 | -0.019 | 1.000 |
| i120 MaxNT - i20 MeanCountT | 0.283 | 0.555 | 431 | 0.510 | 1.000 |
| i120 MaxNT - i120 SpeciesRichness | 0.025 | 0.555 | 431 | 0.045 | 1.000 |
| i180 MaxNT - i180 MeanCountT | 1.990 | 0.555 | 431 | 3.585 | **0.064** |
| i180 MaxNT - i180 SpeciesRichness | 0.351 | 0.555 | 431 | 0.631 | 1.000 |
| i360 MaxNT - i360 MeanCountT | 1.395 | 0.555 | 431 | 2.514 | 0.665 |
| i360 MaxNT - i360 SpeciesRichness | 0.214 | 0.555 | 431 | 0.385 | 1.000 |
| i12 MeanCountT - i12 SpeciesRichness | -2.134 | 0.555 | 431 | -3.845 | **0.027** |
| i15 MeanCountT - i15 SpeciesRichness | -2.228 | 0.555 | 431 | -4.013 | **0.015** |
| i20 MeanCountT - i20 SpeciesRichness | -2.325 | 0.555 | 431 | -4.189 | **0.008** |
| i30 MeanCountT - i30 SpeciesRichness | -2.690 | 0.555 | 431 | -4.846 | **<.001** |
| i60 MeanCountT - i60 SpeciesRichness | -2.335 | 0.555 | 431 | -4.207 | **0.007** |
| i120 MeanCountT - i120 SpeciesRichness | -2.055 | 0.555 | 431 | -3.702 | **0.044** |
| i180 MeanCountT - i180 SpeciesRichness | -1.640 | 0.555 | 431 | -2.954 | 0.329 |
| i360 MeanCountT - i360 SpeciesRichness | -1.182 | 0.555 | 431 | -2.129 | 0.901 |
