# Supplementary material for: Optimising sampling of fish assemblages on intertidal reefs using remote underwater video
Source: PeerJ. 2023 May 22;11:e15426. doi: 10.7717/peerj.15426 (PMC10211360; doi:10.7717/peerj.15426)
Supplement: Supplemental Information 4 [file peerj-11-15426-s004.docx]

| **Contrast** | **estimate** | **SE** | **df** | **t.ratio** | **p.value** |
| --- | --- | --- | --- | --- | --- |
| i12 MaxN - i12 MeanCount | 3.890 | 0.317 | 287 | 12.258 | **<.001** |
| i15 MaxN - i15 MeanCount | 3.901 | 0.317 | 287 | 12.292 | **<.001** |
| i20 MaxN - i20 MeanCount | 4.134 | 0.317 | 287 | 13.029 | **<.001** |
| i30 MaxN - i30 MeanCount | 4.040 | 0.317 | 287 | 12.73 | **<.001** |
| i60 MaxN - i60 MeanCount | 3.828 | 0.317 | 287 | 12.064 | **<.001** |
| i120 MaxN - i120 MeanCount | 3.403 | 0.317 | 287 | 10.723 | **<.001** |
| i180 MaxN - i180 MeanCount | 3.365 | 0.317 | 287 | 10.604 | **<.001** |
| i360 MaxN - i360 MeanCount | 2.534 | 0.317 | 287 | 7.985 | **<.001** |
