# Supplementary material for: Optimising sampling of fish assemblages on intertidal reefs using remote underwater video
Source: PeerJ. 2023 May 22;11:e15426. doi: 10.7717/peerj.15426 (PMC10211360; doi:10.7717/peerj.15426)
Supplement: Supplemental Information 5 [file peerj-11-15426-s005.docx]

| **Contrast** | **estimate** | **SE** | **df** | **t.ratio** | **p.value** |
| --- | --- | --- | --- | --- | --- |
| i = 12: | | | | | |
| Random MaxNT - Systematic MaxNT | -0.433 | 0.377 | 430 | -1.150 | 1 |
| Random MaxNT - Random MeanCountT | 0.030 | 0.387 | 430 | 0.077 | 1 |
| Random MaxNT - Systematic MeanCountT | 0.053 | 0.388 | 430 | 0.137 | 1 |
| Random MaxNT - Random SpeciesRichness | 0.588 | 0.401 | 430 | 1.465 | 1 |
| Random MaxNT - Systematic SpeciesRichness | 0.511 | 0.399 | 430 | 1.281 | 1 |
| Systematic MaxNT - Random MeanCountT | 0.463 | 0.377 | 430 | 1.227 | 1 |
| Systematic MaxNT - Systematic MeanCountT | 0.486 | 0.378 | 430 | 1.287 | 1 |
| Systematic MaxNT - Random SpeciesRichness | 1.021 | 0.392 | 430 | 2.606 | 0.142 |
| Systematic MaxNT - Systematic SpeciesRichness | 0.944 | 0.390 | 430 | 2.424 | 0.237 |
| Random MeanCountT - Systematic MeanCountT | 0.023 | 0.388 | 430 | 0.060 | 1 |
| Random MeanCountT - Random SpeciesRichness | 0.558 | 0.402 | 430 | 1.388 | 1 |
| Random MeanCountT - Systematic SpeciesRichness | 0.481 | 0.400 | 430 | 1.204 | 1 |
| Systematic MeanCountT - Random SpeciesRichness | 0.534 | 0.402 | 430 | 1.329 | 1 |
| Systematic MeanCountT - Systematic SpeciesRichness | 0.458 | 0.400 | 430 | 1.144 | 1 |
| Random SpeciesRichness - Systematic SpeciesRichness | -0.077 | 0.413 | 430 | -0.185 | 1 |
|  |  |  |  |  |  |
| i = 15: | | | | | |
| Random MaxNT - Systematic MaxNT | 0.44 | 0.380 | 430 | 1.158 | 1 |
| Random MaxNT - Random MeanCountT | 0.748 | 0.387 | 430 | 1.932 | 0.811 |
| Random MaxNT - Systematic MeanCountT | 0.885 | 0.391 | 430 | 2.265 | 0.36 |
| Random MaxNT - Random SpeciesRichness | 0.970 | 0.393 | 430 | 2.468 | 0.21 |
| Random MaxNT - Systematic SpeciesRichness | 1.149 | 0.398 | 430 | 2.887 | 0.061 |
| Systematic MaxNT - Random MeanCountT | 0.309 | 0.397 | 430 | 0.777 | 1 |
| Systematic MaxNT - Systematic MeanCountT | 0.446 | 0.401 | 430 | 1.113 | 1 |
| Systematic MaxNT - Random SpeciesRichness | 0.531 | 0.403 | 430 | 1.317 | 1 |
| Systematic MaxNT - Systematic SpeciesRichness | 0.709 | 0.408 | 430 | 1.740 | 1 |
| Random MeanCountT - Systematic MeanCountT | 0.137 | 0.408 | 430 | 0.336 | 1 |
| Random MeanCountT - Random SpeciesRichness | 0.222 | 0.410 | 430 | 0.541 | 1 |
| Random MeanCountT - Systematic SpeciesRichness | 0.401 | 0.415 | 430 | 0.966 | 1 |
| Systematic MeanCountT - Random SpeciesRichness | 0.085 | 0.414 | 430 | 0.205 | 1 |
| Systematic MeanCountT - Systematic SpeciesRichness | 0.264 | 0.418 | 430 | 0.631 | 1 |
| Random SpeciesRichness - Systematic SpeciesRichness | 0.179 | 0.420 | 430 | 0.426 | 1 |
|  |  |  |  |  |  |
| i = 20: | | | | | |
| Random MaxNT - Systematic MaxNT | 0.159 | 0.382 | 430 | 0.417 | 1 |
| Random MaxNT - Random MeanCountT | 0.551 | 0.391 | 430 | 1.407 | 1 |
| Random MaxNT - Systematic MeanCountT | 0.680 | 0.395 | 430 | 1.724 | 1 |
| Random MaxNT - Random SpeciesRichness | 0.855 | 0.399 | 430 | 2.142 | 0.492 |
| Random MaxNT - Systematic SpeciesRichness | 0.854 | 0.399 | 430 | 2.138 | 0.496 |
| Systematic MaxNT - Random MeanCountT | 0.391 | 0.395 | 430 | 0.990 | 1 |
| Systematic MaxNT - Systematic MeanCountT | 0.521 | 0.398 | 430 | 1.308 | 1 |
| Systematic MaxNT - Random SpeciesRichness | 0.696 | 0.403 | 430 | 1.728 | 1 |
| Systematic MaxNT - Systematic SpeciesRichness | 0.694 | 0.403 | 430 | 1.724 | 1 |
| Random MeanCountT - Systematic MeanCountT | 0.130 | 0.408 | 430 | 0.319 | 1 |
| Random MeanCountT - Random SpeciesRichness | 0.305 | 0.412 | 430 | 0.740 | 1 |
| Random MeanCountT - Systematic SpeciesRichness | 0.303 | 0.412 | 430 | 0.736 | 1 |
| Systematic MeanCountT - Random SpeciesRichness | 0.175 | 0.415 | 430 | 0.421 | 1 |
| Systematic MeanCountT - Systematic SpeciesRichness | 0.173 | 0.415 | 430 | 0.417 | 1 |
| Random SpeciesRichness - Systematic SpeciesRichness | -0.002 | 0.420 | 430 | -0.004 | 1 |
|  |  |  |  |  |  |
| i = 30: | | | | | |
| Random MaxNT - Systematic MaxNT | -0.028 | 0.389 | 430 | -0.072 | 1 |
| Random MaxNT - Random MeanCountT | 0.551 | 0.404 | 430 | 1.365 | 1 |
| Random MaxNT - Systematic MeanCountT | 0.967 | 0.415 | 430 | 2.329 | 0.305 |
| Random MaxNT - Random SpeciesRichness | 0.760 | 0.409 | 430 | 1.856 | 0.961 |
| Random MaxNT - Systematic SpeciesRichness | 0.899 | 0.413 | 430 | 2.175 | 0.453 |
| Systematic MaxNT - Random MeanCountT | 0.579 | 0.403 | 430 | 1.437 | 1 |
| Systematic MaxNT - Systematic MeanCountT | 0.995 | 0.415 | 430 | 2.401 | 0.252 |
| Systematic MaxNT - Random SpeciesRichness | 0.788 | 0.409 | 430 | 1.928 | 0.818 |
| Systematic MaxNT - Systematic SpeciesRichness | 0.927 | 0.413 | 430 | 2.246 | 0.378 |
| Random MeanCountT - Systematic MeanCountT | 0.416 | 0.428 | 430 | 0.971 | 1 |
| Random MeanCountT - Random SpeciesRichness | 0.209 | 0.423 | 430 | 0.494 | 1 |
| Random MeanCountT - Systematic SpeciesRichness | 0.348 | 0.426 | 430 | 0.815 | 1 |
| Systematic MeanCountT - Random SpeciesRichness | -0.207 | 0.433 | 430 | -0.478 | 1 |
| Systematic MeanCountT - Systematic SpeciesRichness | -0.068 | 0.437 | 430 | -0.156 | 1 |
| Random SpeciesRichness - Systematic SpeciesRichness | 0.139 | 0.432 | 430 | 0.322 | 1 |
|  |  |  |  |  |  |
| i = 60: | | | | | |
| Random MaxNT - Systematic MaxNT | 0.605 | 0.402 | 430 | 1.503 | 1 |
| Random MaxNT - Random MeanCountT | 0.663 | 0.404 | 430 | 1.642 | 1 |
| Random MaxNT - Systematic MeanCountT | 1.490 | 0.428 | 430 | 3.486 | **0.008** |
| Random MaxNT - Random SpeciesRichness | 0.948 | 0.412 | 430 | 2.303 | 0.326 |
| Random MaxNT - Systematic SpeciesRichness | 1.253 | 0.402 | 430 | 2.981 | **0.046** |
| Systematic MaxNT - Random MeanCountT | 0.059 | 0.418 | 430 | 0.140 | 1 |
| Systematic MaxNT - Systematic MeanCountT | 0.886 | 0.441 | 430 | 2.007 | 0.681 |
| Systematic MaxNT - Random SpeciesRichness | 0.343 | 0.426 | 430 | 0.806 | 1 |
| Systematic MaxNT - Systematic SpeciesRichness | 0.648 | 0.434 | 430 | 1.493 | 1 |
| Random MeanCountT - Systematic MeanCountT | 0.827 | 0.443 | 430 | 1.868 | 0.936 |
| Random MeanCountT - Random SpeciesRichness | 0.285 | 0.427 | 430 | 0.667 | 1 |
| Random MeanCountT - Systematic SpeciesRichness | 0.590 | 0.436 | 430 | 1.354 | 1 |
| Systematic MeanCountT - Random SpeciesRichness | -0.542 | 0.450 | 430 | -1.206 | 1 |
| Systematic MeanCountT - Systematic SpeciesRichness | -0.237 | 0.458 | 430 | -0.518 | 1 |
| Random SpeciesRichness - Systematic SpeciesRichness | 0.305 | 0.443 | 430 | 0.688 | 1 |
|  |  |  |  |  |  |
| i = 120: | | | | | |
| Random MaxNT - Systematic MaxNT | 0.222 | 0.428 | 430 | 0.520 | 1 |
| Random MaxNT - Random MeanCountT | 0.714 | 0.442 | 430 | 1.616 | 1 |
| Random MaxNT - Systematic MeanCountT | 1.357 | 0.461 | 430 | 2.941 | 0.052 |
| Random MaxNT - Random SpeciesRichness | 0.521 | 0.436 | 430 | 1.195 | 1 |
| Random MaxNT - Systematic SpeciesRichness | 0.608 | 0.439 | 430 | 1.386 | 1 |
| Systematic MaxNT - Random MeanCountT | 0.491 | 0.447 | 430 | 1.099 | 1 |
| Systematic MaxNT - Systematic MeanCountT | 1.135 | 0.467 | 430 | 2.431 | 0.232 |
| Systematic MaxNT - Random SpeciesRichness | 0.299 | 0.442 | 430 | 0.677 | 1 |
| Systematic MaxNT - Systematic SpeciesRichness | 0.386 | 0.444 | 430 | 0.868 | 1 |
| Random MeanCountT - Systematic MeanCountT | 0.643 | 0.479 | 430 | 1.342 | 1 |
| Random MeanCountT - Random SpeciesRichness | -0.192 | 0.455 | 430 | -0.423 | 1 |
| Random MeanCountT - Systematic SpeciesRichness | -0.106 | 0.457 | 430 | -0.231 | 1 |
| Systematic MeanCountT - Random SpeciesRichness | -0.836 | 0.474 | 430 | -1.762 | 1 |
| Systematic MeanCountT - Systematic SpeciesRichness | -0.749 | 0.477 | 430 | -1.572 | 1 |
| Random SpeciesRichness - Systematic SpeciesRichness | 0.087 | 0.452 | 430 | 0.192 | 1 |
|  |  |  |  |  |  |
| i = 180: | | | | | |
| Random MaxNT - Systematic MaxNT | 0.407 | 0.434 | 430 | 0.937 | 1 |
| Random MaxNT - Random MeanCountT | 0.641 | 0.440 | 430 | 1.455 | 1 |
| Random MaxNT - Systematic MeanCountT | 1.948 | 0.483 | 430 | 4.036 | **0.001** |
| Random MaxNT - Random SpeciesRichness | 0.737 | 0.443 | 430 | 1.662 | 1 |
| Random MaxNT - Systematic SpeciesRichness | 0.82 | 0.446 | 430 | 1.841 | 0.995 |
| Systematic MaxNT - Random MeanCountT | 0.234 | 0.451 | 430 | 0.520 | 1 |
| Systematic MaxNT - Systematic MeanCountT | 1.542 | 0.492 | 430 | 3.132 | **0.028** |
| Systematic MaxNT - Random SpeciesRichness | 0.330 | 0.454 | 430 | 0.728 | 1 |
| Systematic MaxNT - Systematic SpeciesRichness | 0.414 | 0.456 | 430 | 0.907 | 1 |
| Random MeanCountT - Systematic MeanCountT | 1.308 | 0.498 | 430 | 2.625 | 0.135 |
| Random MeanCountT - Random SpeciesRichness | 0.096 | 0.460 | 430 | 0.208 | 1 |
| Random MeanCountT - Systematic SpeciesRichness | 0.179 | 0.462 | 430 | 0.388 | 1 |
| Systematic MeanCountT - Random SpeciesRichness | -1.212 | 0.501 | 430 | -2.421 | 0.239 |
| Systematic MeanCountT - Systematic SpeciesRichness | -1.128 | 0.503 | 430 | -2.243 | 0.381 |
| Random SpeciesRichness - Systematic SpeciesRichness | 0.084 | 0.465 | 430 | 0.180 | 1 |
|  |  |  |  |  |  |
| i = 360: | | | | | |
| Random MaxNT - Systematic MaxNT | 0.661 | 0.459 | 430 | 1.441 | 1 |
| Random MaxNT - Random MeanCountT | 0.791 | 0.463 | 430 | 1.710 | 1 |
| Random MaxNT - Systematic MeanCountT | 2.208 | 0.512 | 430 | 4.311 | **<.001** |
| Random MaxNT - Random SpeciesRichness | 0.572 | 0.456 | 430 | 1.255 | 1 |
| Random MaxNT - Systematic SpeciesRichness | 0.652 | 0.458 | 430 | 1.422 | 1 |
| Systematic MaxNT - Random MeanCountT | 0.130 | 0.480 | 430 | 0.271 | 1 |
| Systematic MaxNT - Systematic MeanCountT | 1.547 | 0.528 | 430 | 2.928 | 0.054 |
| Systematic MaxNT - Random SpeciesRichness | -0.089 | 0.474 | 430 | -0.187 | 1 |
| Systematic MaxNT - Systematic SpeciesRichness | -0.009 | 0.476 | 430 | -0.019 | 1 |
| Random MeanCountT - Systematic MeanCountT | 1.417 | 0.532 | 430 | 2.664 | 0.120 |
| Random MeanCountT - Random SpeciesRichness | -0.219 | 0.478 | 430 | -0.458 | 1 |
| Random MeanCountT - Systematic SpeciesRichness | -0.139 | 0.480 | 430 | -0.290 | 1 |
| Systematic MeanCountT - Random SpeciesRichness | -1.636 | 0.526 | 430 | -3.109 | **0.030** |
| Systematic MeanCountT - Systematic SpeciesRichness | -1.556 | 0.528 | 430 | -2.947 | 0.051 |
| Random SpeciesRichness - Systematic SpeciesRichness | 0.080 | 0.474 | 430 | 0.168 | 1 |
