# Supplementary material for: Optimising sampling of fish assemblages on intertidal reefs using remote underwater video
Source: PeerJ. 2023 May 22;11:e15426. doi: 10.7717/peerj.15426 (PMC10211360; doi:10.7717/peerj.15426)
Supplement: Supplemental Information 6 [file peerj-11-15426-s006.docx]

| **Contrast** | **estimate** | **SE** | **df** | **t.ratio** | **p.value** |  |
| --- | --- | --- | --- | --- | --- | --- |
| i=12: | | | | | | |
| RandomMaxN-SystematicMaxN | -0.031 | 0.254 | 286 | -0.123 | 1 |  |
| RandomMaxN-RandomMeanCount | 0.065 | 0.256 | 286 | 0.252 | 1 |  |
| RandomMaxN-SystematicMeanCount | 0.256 | 0.260 | 286 | 0.982 | 1 |  |
| SystematicMaxN-RandomMeanCount | 0.096 | 0.255 | 286 | 0.375 | 1 |  |
| SystematicMaxN-SystematicMeanCount | 0.287 | 0.260 | 286 | 1.105 | 1 |  |
| RandomMeanCount-SystematicMeanCount | 0.191 | 0.262 | 286 | 0.731 | 1 |  |
|  |  |  |  |  |  |  |
| i=15: | | | | | | |
| RandomMaxN-SystematicMaxN | 0.144 | 0.256 | 286 | 0.562 | 1 |  |
| RandomMaxN-RandomMeanCount | 0.349 | 0.260 | 286 | 1.340 | 1 |  |
| RandomMaxN-SystematicMeanCount | 0.465 | 0.263 | 286 | 1.767 | 0.470 |  |
| SystematicMaxN-RandomMeanCount | 0.205 | 0.263 | 286 | 0.779 | 1 |  |
| SystematicMaxN-SystematicMeanCount | 0.321 | 0.266 | 286 | 1.206 | 1 |  |
| RandomMeanCount-SystematicMeanCount | 0.116 | 0.270 | 286 | 0.428 | 1 |  |
|  |  |  |  |  |  |  |
| i=20: | | | | | | |
| RandomMaxN-SystematicMaxN | -0.036 | 0.264 | 286 | -0.137 | 1 |  |
| RandomMaxN-RandomMeanCount | 0.280 | 0.271 | 286 | 1.032 | 1 |  |
| RandomMaxN-SystematicMeanCount | 0.147 | 0.268 | 286 | 0.547 | 1 |  |
| SystematicMaxN-RandomMeanCount | 0.316 | 0.271 | 286 | 1.169 | 1 |  |
| SystematicMaxN-SystematicMeanCount | 0.183 | 0.268 | 286 | 0.684 | 1 |  |
| RandomMeanCount-SystematicMeanCount | -0.133 | 0.275 | 286 | -0.485 | 1 |  |
|  |  |  |  |  |  |  |
| i=30: | | | | | | |
| RandomMaxN-SystematicMaxN | 0.040 | 0.263 | 286 | 0.151 | 1 |  |
| RandomMaxN-RandomMeanCount | 0.279 | 0.268 | 286 | 1.038 | 1 |  |
| RandomMaxN-SystematicMeanCount | 0.665 | 0.278 | 286 | 2.396 | 0.103 |  |
| SystematicMaxN-RandomMeanCount | 0.239 | 0.269 | 286 | 0.887 | 1 |  |
| SystematicMaxN-SystematicMeanCount | 0.625 | 0.278 | 286 | 2.246 | 0.153 |  |
| RandomMeanCount-SystematicMeanCount | 0.387 | 0.284 | 286 | 1.364 | 1 |  |
|  |  |  |  |  |  |  |
| i=60: | | | | | | |
| RandomMaxN-SystematicMaxN | 0.259 | 0.280 | 286 | 0.924 | 1 |  |
| RandomMaxN-RandomMeanCount | 0.351 | 0.283 | 286 | 1.243 | 1 |  |
| RandomMaxN-SystematicMeanCount | 0.907 | 0.297 | 286 | 3.054 | **0.015** |  |
| SystematicMaxN-RandomMeanCount | 0.092 | 0.288 | 286 | 0.320 | 1 |  |
| SystematicMaxN-SystematicMeanCount | 0.649 | 0.303 | 286 | 2.143 | 0.198 |  |
| RandomMeanCount-SystematicMeanCount | 0.556 | 0.305 | 286 | 1.825 | 0.414 |  |
|  |  |  |  |  |  |  |
| i=120: | | | | | | |
| RandomMaxN-SystematicMaxN | 0.121 | 0.291 | 286 | 0.415 | 1 |  |
| RandomMaxN-RandomMeanCount | 0.564 | 0.302 | 286 | 1.866 | 0.378 |  |
| RandomMaxN-SystematicMeanCount | 1.031 | 0.316 | 286 | 3.265 | **0.007** |  |
| SystematicMaxN-RandomMeanCount | 0.443 | 0.305 | 286 | 1.453 | 0.883 |  |
| SystematicMaxN-SystematicMeanCount | 0.910 | 0.318 | 286 | 2.858 | **0.027** |  |
| RandomMeanCount-SystematicMeanCount | 0.467 | 0.329 | 286 | 1.419 | 0.942 |  |
|  |  |  |  |  |  |  |
| i=180: | | | | | | |
| RandomMaxN-SystematicMaxN | 0.054 | 0.304 | 286 | 0.177 | 1 |  |
| RandomMaxN-RandomMeanCount | 0.477 | 0.315 | 286 | 1.512 | 0.790 |  |
| RandomMaxN-SystematicMeanCount | 1.541 | 0.349 | 286 | 4.412 | **<0.001** |  |
| SystematicMaxN-RandomMeanCount | 0.423 | 0.317 | 286 | 1.336 | 1 |  |
| SystematicMaxN-SystematicMeanCount | 1.487 | 0.350 | 286 | 4.243 | **<0.001** |  |
| RandomMeanCount-SystematicMeanCount | 1.064 | 0.360 | 286 | 2.953 | **0.020** |  |
|  |  |  |  |  |  |  |
| i=160: | | | | | | |
| RandomMaxN-SystematicMaxN | -0.435 | 0.322 | 286 | -1.350 | 1 |  |
| RandomMaxN-RandomMeanCount | 0.192 | 0.338 | 286 | 0.567 | 1 |  |
| RandomMaxN-SystematicMeanCount | 1.408 | 0.379 | 286 | 3.716 | **0.002** |  |
| SystematicMaxN-RandomMeanCount | 0.626 | 0.327 | 286 | 1.913 | 0.340 |  |
| SystematicMaxN-SystematicMeanCount | 1.843 | 0.369 | 286 | 4.993 | **<0.001** |  |
| RandomMeanCount-SystematicMeanCount | 1.216 | 0.384 | 286 | 3.170 | **0.010** |  |
